# Measurement of diets that are healthy, environmentally sustainable, affordable, and equitable: A scoping review of metrics, findings, and research gaps

**DOI:** 10.3389/fnut.2023.1125955

**Published:** 2023-04-03

**Authors:** Patrick Webb, Kara Livingston Staffier, Hyomin Lee, Brian Howell, Kyra Battaglia, Brooke M. Bell, Julia Matteson, Nicola M. McKeown, Sean B. Cash, Fang Fang Zhang, Jessica L. Decker Sparks, Nicole Tichenor Blackstone

**Affiliations:** ^1^Gerald J. and Dorothy R. Friedman School of Nutrition Science and Policy, Tufts University, Boston, MA, United States; ^2^Nutritional Epidemiology Program, Jean Mayer United States Department of Agriculture Human Nutrition Research Center on Aging, Tufts University, Boston, MA, United States; ^3^Department of Health Science, Sargent College of Health and Rehabilitation Sciences, Boston University, Boston, MA, United States

**Keywords:** dietary patterns, health, climate, environment, affordability, equity, evidence synthesis

## Abstract

**Introduction:**

Research on the impacts of dietary patterns on human and planetary health is a rapidly growing field. A wide range of metrics, datasets, and analytical techniques has been used to explore the role of dietary choices/constraints in driving greenhouse gas (GHG) emissions, environmental degradation, health and disease outcomes, and the affordability of food baskets. Many argue that each domain is important, but few have tackled all simultaneously in analyzing diet-outcome relationships.

**Methods:**

This paper reviews studies published between January 2015 and December 2021 (inclusive) that examined dietary patterns in relation to at least two of the following four thematic pillars: (i) planetary health, including, climate change, environmental quality, and natural resource impacts, (ii) human health and disease, (iii) economic outcomes, including diet cost/affordability, and (iv) social outcomes, e.g., wages, working conditions, and culturally relevant diets. We systematically screened 2,425 publications by title and abstract and included data from 42 eligible publications in this review.

**Results:**

Most dietary patterns used were statistically estimated or simulated rather than observed. A rising number of studies consider the cost/affordability of dietary scenarios in relation to optimized environmental and health outcomes. However, only six publications incorporate social sustainability outcomes, which represents an under-explored dimension of food system concerns.

**Discussion:**

This review suggests a need for (i) transparency and clarity in datasets used and analytical methods; (ii) explicit integration of indicators and metrics linking social and economic issues to the commonly assessed diet-climate-planetary ecology relationships; (iii) inclusion of data and researchers from low- and middle-income countries; (iv) inclusion of processed food products to reflect the reality of consumer choices globally; and (v) attention to the implications of findings for policymakers. Better understanding is urgently needed on dietary impacts on all relevant human and planetary domains simultaneously.

## 1. Introduction

A rapidly expanding literature explores linkages among dietary patterns and a range of human and planetary health outcomes. This body of research informs ongoing dialogues regarding policy-level actions and investments needed at national and global levels to meet not only climate-related emissions targets, but equally to tackle malnutrition in all its forms, support “nature positive” actions that will preserve vital ecosystem services, and to promote a just transition from exploitative to inclusive food systems governance (1, 2). At the interface of these key challenges is the quality of people’s diets. What individuals eat, or are unable to eat, and how their foods are produced and processed matters to many human health outcomes, but the wide economic and societal processes that underpin and shape diets also have characteristics that impact negatively on planetary health *via*, for example, greenhouse gas (GHG) emissions natural resource depletion and biodiversity loss (3, 4).

Attempts to model such linkages, and to quantify the scale and direction of such dynamic interactions, have accumulated at a lively pace in the past decade and continue to expand the evidence base in important ways. In recent years, several important developments have moved this field of study forward. First, early analyses based on single issue pairings, such as diets and climate change, or diets and diseases, have given way to more complex modeling that integrates multiple interactions across multiple exposures and outcomes of interest. Second, a widening range of climate and environment indicators has been incorporated into modeling, moving quickly from land use conversion, water extraction and species extinction to indicators such as ozone layer depletion (5), and particulate air pollution (6) to per capita non-renewable energy use (7). Third, metrics of human health and nutrition outcomes have become more diverse as the evidence base underpinning diet-disease relationships has expanded and deepened, moving from a predominant focus on mortality from cardiovascular disease and cancer (8) to deaths avoided from type 2 diabetes mellitus (9), or even prevalence of serum retinol deficiency (10).

That said, while the proliferation of food systems-diet-climate-health research brings increasing rigor and nuance to what are very complex planet-wide challenges, many gaps remain in our understanding of the most important multi-system interactions influenced by dietary patterns. This is particularly true of the social and economic dimensions of food systems functions. In 2010, the Food and Agriculture Organization of the United Nations (FAO) defined sustainable diets as “ones that support human health, have low environmental impacts, and are also affordable and culturally acceptable” (11). In other words, economic and social dimensions were identified as important to the overall sustainability of food systems as the environment and health domains.

Despite this recognition, a 2015 review of the emerging literature argued a need for integrative assessments of the environmental, social, and economic impacts of foods and diets (12). More reviews published since 2015 still called for fuller incorporation of social and economic parameters in diet-climate-environment-health modeling. Too much reliance has been placed on environmental and health assessments of diets, failing to accurately encapsulate the economic and social dimensions (13, 14). The lack of representation of the social pillar can be partially attributed to lack of data and metrics, as well as limited understanding of how best to define social sustainability (14). To fully understand the true trade-offs that can result from dietary shifts, research must begin to incorporate all four pillars of sustainability, with specific attention on economic and social implications (15, 16).

Since the initial calls for action over half a decade ago, progress has been slow. While there is growing use in publications of terms like social justice, just transitions, diet disparities and socio-cultural acceptability (17–19), the incorporation of such issues into food-climate-health simulations and modeling is still nascent. One recent review of how nutrition, health, and agriculture researchers deal with equity issues concluded that while most studies have considered inequity as a concept, the underlying drivers of inequity from a social, economic, environmental, and/or health standpoint continue to be understudied (20). Indeed, research needs to consider systemic factors of inequity through a dietary lens.

This paper systematically reviews literature published between 2015 and 2021 to determine (i) how many studies include social and economic indicators in their analysis of dietary patterns impacts on human and planetary health, (ii) what analytical approaches, methods, and metrics are used to address social and economic concerns when these are included, and (iii) what conceptual and empirical gaps persist that represent impediments to meaningful target-setting and policy change.

## 2. Background

It is widely accepted that today’s food systems negatively impact the ecological foundations on which food production relies, including land, water, biodiversity, etc. (21, 22). It is also acknowledged that food system functions simultaneously generate significant GHG emissions that contribute to climate change, while underpinning dietary patterns associated with a global escalation of diet-related non-communicable diseases while leaving millions of other people undernourished and allowing food system worker exploitation to persist (23, 24). This has led to calls for food (or agri-food) system transformation, on the one hand, and for shifts in dietary patterns to facilitate such transformation, on the other hand (25). Most of the recommended dietary shifts are based on assessments of the degree to which resulting food demand could simultaneously reduce climate emissions and other environmental impacts, while improving human health outcomes, typically at the global level. Other reference diets have been crafted at the national level, using additional parameters such as consumer acceptability (18), affordability (26) and trade-offs among competing policy priorities (27).

In most studies, authors point to the importance of better defining optimal diets as a means of guiding policymakers in their task of seeking to transform food systems for improved and sustained human and planetary health (28, 29). That said, designing and implementing effective food system-wide transformation will require the use of a comprehensive set of metrics that are meaningful across all sectoral activities from production through to consumption. But there is a lack of consensus on what transformation should entail, and on what the endpoint(s) should look like. As a result, there is continued debate regarding what indicators should be used to establish meaningful goals, track food system transformation and performance, and assess the net impacts of food system interventions (30).

The suite of indicators used for modeling of climate and environmental dynamics is relatively standardized in relation to accepted concepts of planetary boundaries, although the number and type of indicators used continues to grow as new data become available (31). The most common environment-related metrics include GHG emissions, water extraction, land use changes that release carbon to the atmosphere, and loss of biodiversity.

The same is true of metrics that characterize human health outcomes. Although there has recently been a proliferation of indicators used in modeling diet and health (32), most focus on specific dietary exposures in relation to a small number of outcomes, such as all-cause mortality risk (33), Disability-Adjusted Life Years (34), or diet-related non-communicable diseases like cardiovascular disease (CVD), certain cancers, and type 2 diabetes (T2D) (35). Some studies characterize specific diets that are deemed to be healthful – fulfilling all or most nutritional recommendations in dietary guidelines, such as the Mediterranean Diet which has been discussed since the 1960s (36) and/or sensitive to environmental sustainability concerns (such as the Eat-Lancet Planetary Health Diet, described in (2)), and compare those with prevailing patterns of consumption.

By contrast, the inclusion of measures on economic dimensions is relatively recent and tends to be narrowly framed. The conceptualization of food system issues through a socioeconomic lens predates FAO’s 2010 definition of “sustainable diets.” The Brundtland Report of 1987 was among the first initiatives to call for a systemic perspective that binds environmental with social and economic sustainability concerns (37). The latter report posited that human actions and the environment are synergistic. Similarly, the affordability of food was explicitly mentioned as a core aspect of sustainable diets as early as 2012, yet food price, affordability of recommended dietary patterns, and the distributional impacts of dietary shifts received limited analytical attention until recently. Hirvonen et al. (26), for example, considered the affordability of the Planetary Health Diet using local market prices and household income across 159 countries. The authors concluded that the cost of the reference diet – a basket of foods – would exceed total household income for around 1.58 billion people. The latter projected outcome would, however, be in the absence of any large-scale policy interventions aimed at bolstering the purchasing power of poorest households, such as income transfers *via* social safety nets, and/or taxes and subsidies that could shift the relative prices of foods to support demand for nutrient-dense food items. Policies and targeted interventions can be modifiers of projected outcomes, but these are insufficiently included in most modeling exercises because they would by design impact the equilibrium in prices, consumer demand, and producer investments that are assumed to be stable in most studies.

The social dimension of diet-environment-health relationships is also under-represented in the current literature and has been limited to some socioeconomic indicators (14). These indicators are typically poorly characterized, defined, or validated. Conceptual constructs like equity, social values, child labor, social justice, gender equality, solidarity networks, farmworker justice, agency, dignity in food traditions, animal welfare, and inclusivity, represent important additions to the discussion of potential impacts of dietary and food system transitions (7, 38–42). Indeed, the whole question of food system transformation must be framed by social and economic concerns, and take into consideration perspectives of multiple stakeholders, including consumers, investors, suppliers, workers, policymakers, all of whom have specific expertise and identities that shape their framing (43).

The recent call for greater attention to such issues reflects their continued absence in much recent research. While the four major dimensions of sustainable healthy diets – planetary health, human health, economic, and social outcomes – have been agreed in broad outline for decades, combining them all into studies of dietary change has represented a challenge that is still to be overcome. This paper explores how much of the recent literature captures and integrates all four dimensions in its analysis and thinking. We review the most recent research that puts dietary patterns at the center of feedback loops linking climate and ecology, human health and nutrition, food prices, and social justice. The paper considers metrics, methodologies and datasets used, as well as constraints identified, gaps in the research agenda, and implications of findings.

## 3. Methods

The reporting of this review adheres to the Preferred Reporting Items for Systematic reviews and Meta-Analyses extension for Scoping Reviews (PRISMA-ScR) checklist (44). A review protocol for this scoping review was not previously published.

### 3.1. Search strategy

Peer-reviewed literature searchable in Web of Science, SCOPUS, and OVID Medline between January 1, 2015 and September 30, 2021 were eligible for inclusion. Searches were re-run in February 2022 to update with recent literature. This search led to the inclusion of 2 additional papers published between September 2021 and December 31, 2021. Identified abstracts were screened applying predefined inclusion and exclusion criteria. The search term strategy used is provided in Supplementary Table A1. An informal search process was also conducted by backward tracking citations found in bibliographies of key articles.

### 3.2. Inclusion criteria

Studies needed to examine at least one dietary pattern in relation to at least two outcomes from distinct sustainability pillars. These outcomes needed to fall under four dimensions of sustainability: (1) environment, (2) health, (3) economic, and (4) social. Table 1 provides the full definitions for dietary patterns, outcomes, and the sustainability pillars – health, environment, social, and economic – used in this paper. Studies also needed to meet the following criteria:

**TABLE 1:** Definitions and conceptual scope of terms used.

Pillars	Definition
Health	Outcomes related to diseases or human wellbeing that are associated with meeting nutrient needs, supporting physiological and cognitive growth and development, and promoting wellness [adapted from Nicholls and Drewnowski (14)].
Environment	The impacts on climate, ecosystems, and natural resources resulting from the production, distribution, consumption, and disposal of food commodities and products that underpin dietary patterns.
Social	The underlying conditions within, and the impacts of food supply chains on, stakeholders who are directly or indirectly affected by food system functions. Stakeholder groups include workers, value chain actors, local communities, consumers, society, and children (45). While the wellbeing of people is most focused-on, the wellbeing of animals is also a concern.
Economic	Outcomes related to economic access by consumers to desired foods, including affordability and relative food prices, as well as the cost of policy actions and the viability of the supply chains and institutions that support all food system functions.
**Conceptual**	
Dietary pattern	“The quantities, proportions, variety, or combination of different foods, drinks, and nutrients in diets, and the frequency with which they are habitually consumed” (46).
Observed or approximated current diets	Diets based on self-reported food consumption data, including 24-h recall, food frequency questionnaires, etc. or food consumption proxy data, including food availability data (e.g., food balance sheets), often at a national level and downscaled to per capita, and food acquisition data, e.g., purchase surveys, often at the household level and adjusted to per capita.
Diet scores or indices	Diet scores or indices used to assess level of adherence to a benchmark (e.g., the Healthy Eating Index).
Dietary pattern archetypes	Reference diet archetypes (USDA Food Patterns, EAT-Lancet pattern, Mediterranean pattern) or patterns that are not necessarily evidence-based and/or may have wide variability in definition, including vegan or flexitarian.
Statistically estimated or modeled diets	Dietary patterns resulting from optimization methods (e.g., least cost diet methods and linear programming); or estimation methods that explain variation in patterns (e.g., factor analysis), aggregate individuals into groups with non-overlapping patterns (e.g., cluster analysis), or project current patterns into the future (e.g., simulation modeling).
Outcome	Outcomes were defined as “endpoints”: variables that were assessed to document the effect of exposure to a dietary pattern or change in dietary pattern. The endpoints represent measured or estimated various forms of positive and/or negative effects under each pillar.

1. Dietary patterns must be characterized either by per-capita food consumption data, food consumption proxy data, or be a simulated estimate of a dietary pattern.
a. Dietary assessment methods may include: food frequency questionnaires, 24-hour recall, food diaries, food availability data, food balance sheets, etc.
2. Dietary patterns must measure and incorporate at least three food items or groups.
a. For examples, studies that only examine the consumption of a single food item or group (e.g., sugar sweetened beverages, fruit and vegetables, meat consumption) were not eligible for inclusion in this review.

### 3.3. Types of studies

Eligible study designs included cross-sectional studies, case-control studies, experimental studies, the latter including uncontrolled, non-randomized controlled, and randomized controlled trials, cohort studies (retrospective and prospective), and optimization modeling studies.

### 3.4. Outcomes

Studies must have examined at least two of four sustainability pillars in relation to an eligible dietary pattern, as described above, or a dietary pattern change, relating to intervention/exposure. For our purposes, outcomes are defined as endpoints, including variables that were assessed to document the effect of exposure to a dietary pattern or change in dietary pattern. The endpoints represent measured or estimated various forms of positive and/or negative effects under each pillar (Table 1). We did not consider covariates, grouping variables, stratification variables, mediators, or moderators as eligible outcomes.

### 3.5. Exclusion criteria

Potentially eligible studies were included if they explicitly examined diets in relation to at least two outcome domains, but not if they only discussed those domains without analyzing them explicitly. For example, a study by Blas et al. (47) that compared current food consumption patterns in Spain relative to the Mediterranean Diet was not included because it assumed environmental and health outcomes from included foods rather than analyze them explicitly. That is, impacts on water consumption were derived (rather than calculated) from estimates of “embedded water content” of individual food items, while health impacts were assumed based on the nutrient content of the different diets. Also excluded were reviews and studies that were published outside the specified date range of 2015-2021, analyses based on selected individual foods or food groups, rather than dietary patterns as a whole, studies that had sample sizes of <100 individuals, and those that considered dietary pattern links to only one domain of interest.

### 3.6. Screening

The screening of abstracts was conducted using Rayyan web and mobile app for systematic reviews (48). At least two independent researchers, under the supervision of PW and NM, screened every title/abstract. Where there was disagreement, the specifics were discussed, and a consensus decision arrived at in consultation with PW and NM. Two independent researchers double screened all full-text articles for abstracts that were accepted as potentially relevant.

**FIGURE 1:**
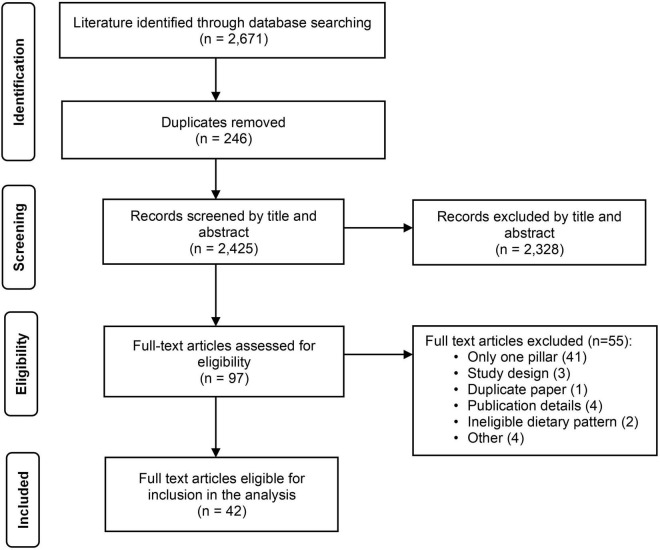
PRISMA flow diagram.

A total of 2,671 publications were retrieved for assessment. After removing duplicates, 2,425 items from published literature searches were screened by title and abstract published in English. After exclusions, 97 full-text articles remained for full paper review. Of those, 42 were eligible for inclusion and are included in the analysis [see Figure 1 adapted from (49)].

### 3.7. Data extraction

The following data were extracted from each publication:

• Publication information, including first author, title, online publication year, and journal name.
• The pillars represented in the article, including health, environment, economic, and social.
• The country or countries from where the data were collected.
• The name of each dietary pattern examined in the article, along with a short description of the pattern and how it was constructed.
• The “typology” of each dietary pattern examined in the article. These typologies were developed *a posteriori* by the research team. Two independent reviewers assigned each dietary pattern to one of four typologies: observed or approximated current diets, diet scores or indices, dietary pattern archetypes, or statistically estimated or modeled diets (see Table 1 for definitions). Coding was based on the description of the diet pattern and how the diets were constructed. Assignment conflicts were resolved by a third reviewer.
• The total number of eligible dietary patterns examined in the article.
• The name of each outcome examined in the article, along with a short description of the outcome and the associated unit of measurement.
• The “category” of each outcome examined in the article. These categories were developed *a posteriori* by the research team. Two independent reviewers assigned each health outcome to one of 10 categories: cancer; cardiovascular diseases; mortality, number of deaths averted, or years of life saved (non-specific disease); type 2 diabetes; stroke; disability-adjusted life year (DALY) (non-specific disease); weight, overweight, or obesity; composite health indicators; quality-adjusted life year (QALY) or quality of life (QOL) related to non-specific diseases; or other. Environment outcomes were assigned to one of 12 categories: climate change, land, water, energy, nitrogen or phosphorus, toxicity, eutrophication, composite environmental indicator, acidification, biodiversity, air pollution, or other. Social outcomes were assigned to one of four categories: acceptability, desirability, food availability, or social risk. Economic outcomes were assigned to one of six categories: food price/cost, economy-level cost, healthcare cost, productivity cost, employment, or other.

The data from each paper was extracted by at least one reviewer, and extraction data was reviewed by another reviewer. Coding was done using tailored spreadsheets in Microsoft^®^ Excel^®^ for Microsoft 365 MSO (Version 2208 Build 16.0.15601.20148) 64-bit. Extraction results were compared and synthesized in Supplementary Files 1–3.

### 3.8. Data synthesis

We used descriptive and comparative analyses to summarize the extracted data items. We calculated the frequencies and percentages for categorical variables, such as the number of dietary patterns, dietary pattern typologies, number of included pillars, and outcome categories, using basic descriptive statistics. Microsoft Excel and R (version 4.1.0) were used to create manuscript figures and tables.

## 4. Results

### 4.1. Study characteristics

**FIGURE 2:**
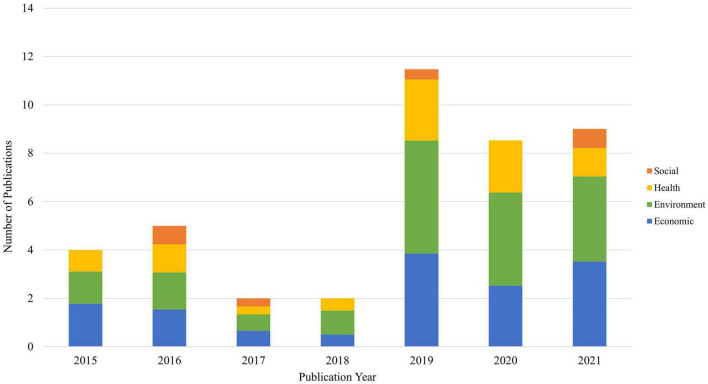
Frequency count of included pillars, by publication year.

Figure 2 shows the pillar breakdown per publication, by online publication year. It does not capture how many outcomes were measured in each pillar, but which pillars each publication covered. The trends show that the majority of included publications (N = 29 of 42, 69.0%) were published between 2019 and 2021. Furthermore, economic, environment, and health outcomes were the most widely represented sustainability pillars measured across all publication years (Figure 2). Social outcomes were rarely examined (N = 6 of 42, 14.3%) and, if included, were present in the most recent years. As will be discussed, no publications included in this review analyzed all four pillars of sustainability simultaneously.

### 4.2. Dietary patterns

From the 42 papers retained for full analysis, a total of 226 dietary patterns were identified, with the vast majority being statistically estimated or simulated diets (65.9%) (Table 2). On average, 5.4 dietary patterns (*SD* = 6.6, min = 1, max = 36) were identified per publication. Nine (21.4%) studies examined one dietary pattern; five (11.9%) studies examined two dietary patterns; four (9.5%) studies examined three dietary patterns; 11 (26.2%) studies examined four dietary patterns; four (9.5%) studies examined five dietary patterns; one (2.4%) study examined six dietary patterns; and eight (19.1%) studies examined seven or more dietary patterns (Supplementary File 2).

**TABLE 2:** Tabulation of dietary pattern typologies included in the final set of studies.

Dietary pattern typology	Frequency (*n*/%)
Statistically estimated or simulated diets	149 (65.9)
Dietary pattern archetype	44 (19.5)
Observed or estimated diets	28 (12.4)
Diet scores or indices	5 (2.2)
Total	226 (100.0)

Dietary patterns were categorized into the four categories identified in Table 2. More recent publications are more consistent in that they typically compare a) an “improved” dietary pattern based on optimization models, national dietary guidelines, or previously published reference diets, with b) “current” dietary patterns usually assessed as an average from national survey data. Nearly half of the publications included in the review (19 of 42, 45.2%) compared an observed or approximated diet (i.e., current diet) to one or more statistically estimated or simulated diets (Supplementary File 2). Six papers (14.3%) compared an observed or approximated diet to one or more dietary pattern archetypes. In other words, researchers tested *a priori* hypotheses either by considering the performance of a given diet pattern type by replacing one food group, like animal-source foods with plant-source foods, or whole dietary patterns with another, or else they used mathematical optimization to model theoretical diets and consider how the food items represented would perform against specified outcomes.

Current dietary patterns were typically constructed as “average” intakes derived from nationally representative or sub-population surveys (Supplementary File 2). The latter were frequently compared with well-known “named” reference diets, such as the Mediterranean Diet, New Nordic diet, or EAT-Lancet Diet, or assessed against recommendations in national dietary guidelines. Also included under current dietary patterns are other unique dietary patterns such as vegetarian, vegan, pescatarian, or flexitarian. However, even “vegetarian” or “flexitarian” diets are defined differently across studies, making like-for-like comparisons difficult without careful attention to the detailed pattern composition.

### 4.3. Pillar outcomes

The starting point for this review was inclusion of dietary patterns with at least two associated sustainability outcomes. That said, more than half of the publications incorporated only two pillars of sustainability: 17 publications (40.5%) examined environment and economic outcomes; five papers (11.9%) examined health and environment outcomes; and one paper (2.4%) examined health and economic outcomes (Supplementary File 1). A smaller proportion included three pillars of sustainability: 13 papers (31.0%) examined health, environment, and economic outcomes; four papers (9.5%) examined environment, economic, and social outcomes; one paper (2.4%) examined health, environment, and social outcomes; and no papers examined health, economic, and social outcomes. There were no publications that incorporated all four pillars of sustainability.

The 42 papers included in this review captured many types of outcomes: 132 related to the environment, 95 related to health, 46 related to economics, and 6 related to social issues (Table 3).

**TABLE 3:** Frequency of outcome measures in each pillar.

Pillar	Outcome measure category	Frequency (*n*/%)
Health	Cancer	22 (23.2)
	Heart-related diseases	20 (21.1)
	Mortality, number of deaths averted, or years of life saved, non-specific disease	15 (15.8)
	Type 2 diabetes	12 (12.6)
	Stroke	10 (10.5)
	Disability-adjusted life year (DALY), non-specific disease	6 (6.3)
	Weight, overweight, or obesity	2 (2.1)
	Quality-adjusted life year (QALY) or quality of life (QOL), non-specific disease	2 (2.1)
	Composite health indicator	1 (1.1)
	*Other*	5 (5.3)
	Total	95 (100.0)
Environment	Climate change	44 (33.3)
	Land	20 (15.2)
	Water	18 (13.6)
	Toxicity	9 (6.8)
	Energy	7 (5.3)
	Eutrophication	7 (5.3)
	Air pollution	6 (4.5)
	Nitrogen or phosphorus	6 (4.5)
	Composite environmental indicator	5 (3.8)
	Acidification	4 (3.0)
	Biodiversity	2 (1.5)
	*Other*	4 (3.0)
	Total	132 (100.0)
Social	Acceptability	2 (33.3)
	Desirability	2 (33.3)
	Food availability	1 (16.7)
	Social risk	1 (16.7)
	Total	6 (100.0)
Economic	Food price/cost	26 (56.5)
	Economy-level cost	7 (15.2)
	Healthcare cost	6 (13.0)
	Productivity cost	3 (6.5)
	Employment	2 (4.3)
	*Other*	2 (4.3)
	Total	46 (100.0)

**FIGURE 3:**
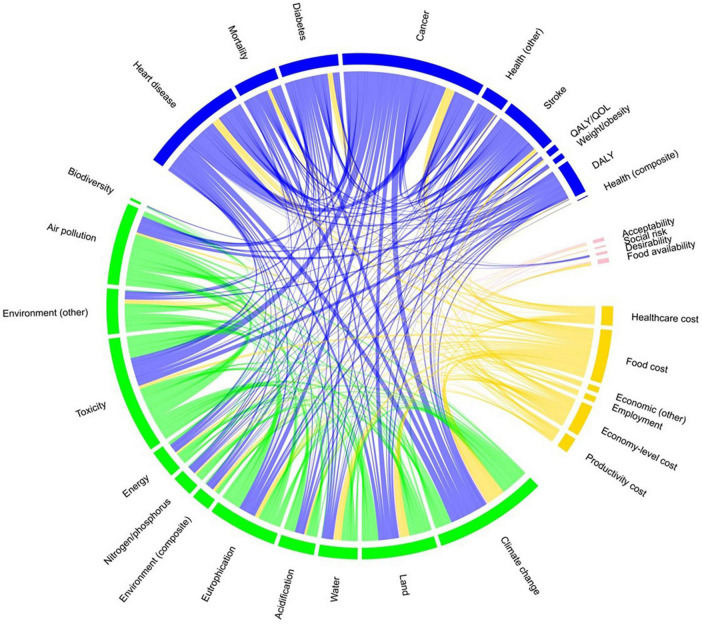
Chord diagram of outcomes.

Figure 3 presents a chord diagram of the links among all the outcomes; that is, which indicators are linked analytically with others in reviewed papers. We found that 81.0% of papers included (N = 34) analyzed at least one environment and one economic outcome. Nineteen papers of 42 total papers (45.2%) examined at least one health and one environment outcome; and 33.3% examined at least one health and one economic outcome.

#### 4.3.1. Environmental outcomes

The most frequently analyzed outcomes focused on the environment domain (n = 132) (Table 3). The environmental indicators that have existed the longest in the literature and have repeatedly been mainly climate-related outcomes, such as GHG emissions (n = 44, 33.3%), land use (n = 20, 15.2%), and water use (n = 18, 13.6%). A smaller number of studies examine toxicity outcomes (n = 9, 6.8%), energy outcomes (n = 7, 5.3%), eutrophication (n = 7, 5.3%), air pollution (n = 6, 4.5%), nitrogen or phosphorus-related outcomes (n = 6, 4.5%), acidification (n = 4, 3.0%), and biodiversity (n = 2, 1.5%).

Most land use metrics used were simple land occupation measures (m2*time), with a small number using more nuanced measures such as cropland scarcity footprints (m2*yr-eq) or dividing land use between cropland and grassland (Supplementary File 3). Water use metrics were more diverse. Eight studies reported water use (blue, fresh, or without modifiers), five studies reported water footprints, two reported blue water footprints (a subset of the water footprint family of metrics; see (50)), and one study each reported water scarcity footprints and water food consumption impacts per dollar spent. The diversity of measures used for land and water outcomes alone demonstrates the challenge of cross-study comparisons in the sustainable diets literature.

Finally, it bears noting that multiple measures we categorized as falling under the environmental pillar also ultimately have human health impacts. These include, but are not limited to, climate change, toxicity, and particulate matter pollution. These outcomes were estimated at the “midpoint level,” using terminology from life cycle assessment (LCA). Midpoint impacts are assessed along the causal chain between the use of resources from the environment or release of emissions to the environment and the final outcomes they cause (51). The LCA framework also allows for estimation of impacts at endpoint or damage level as well, which typically maps to three “areas of protection”: human health, ecosystem quality, and resources. This shows the complexity and interlinkages among the pillars.

#### 4.3.2. Human health outcomes

In the publications included for this review, a total of 95 health and disease-related outcomes were captured (Table 3). The most frequent categories were cancer (n = 22, 23.2%); cardiovascular diseases (n = 20, 21.1%); mortality, number of deaths averted, or number of years of life saved (n = 15, 15.8%); T2D (n = 12, 12.6%); and stroke (N = 10, 10.5%). No studies included outcomes related to nutrient-deficiency diseases or states of undernutrition, which reflects the strong bias in this body of work towards high-income populations. Still lacking altogether are composite metrics that could potentially better reflect overall “health” rather than specific disease outcomes. Our review identified only one composite health indicator (1.1%), the rate advancement period as described in Fresán et al. (52), which incorporates all-cause mortality, cardiovascular disease, breast cancer and type 2 diabetes.

#### 4.3.3. Economic outcomes

The main economic outcomes analyzed were comparing the price (cost) of a reference or optimized diet with prevailing average costs. Although not formally extracted, many publications did consider the distributional effects of price changes associated with dietary shifts, which links to social equity concerns dealt with separately below. A total of 46 economic outcomes were analyzed by included publications: more than half (n = 26, 56.5%) were food price/cost, 15.2% (n = 7) were economy-level outcomes, 13.0% (n = 6) were healthcare costs, 6.5% (n = 3) were productivity costs, and 4.3% (n = 2) were employment-related outcomes (Table 3). Of the 26 papers that analyzed food price and cost, 13 compared current diet costs to the costs of an optimized diet, and 1 compared current diet costs to reference diet costs (Supplementary File 3). Moreover, examples of economy-level outcomes, the second largest economic outcome category analyzed, include gross domestic product (GDP), policy revenue, and policy implementation costs, typically on a national-level scale.

Distributional effects were typically assessed by segmenting populations according to wealth (socioeconomic groups defined by income or expenditure tertiles) and/or race. For example, Arrieta et al. (53), a publication initially included in the extraction, explored six environmental impact indicators associated with two reference diets compared with current consumption patterns in Argentina across ten socioeconomic levels determined by per capita total expenditure. However, it is important to note that in this publication, and all others included in the review, segmentation by socioeconomic status, race, ethnicity, education, etc. is not captured as either a social or economic outcome based on our definitions. Therefore, this paper was not included in the final count of publications as it only assessed outcomes of one pillar.

#### 4.3.4. Social outcomes

This domain has the fewest validated metrics with potential to be fully integrated into modeling and other analyses of dietary impacts. A total of six social outcomes were analyzed across the 42 included papers: two (33.3%) were acceptability outcomes, two (33.3%) were desirability outcomes (relating to taste preferences), one (16.7%) was food availability, and one (16.7%) was social risk (Table 3).

Acceptability measures included a measure of deviations from current diets (54) and a measure of “cultural acceptability” (55). The latter was operationalized as a set of variables used to constrain deviations from current diets (55), including one explicitly cultural variable (i.e., consumption of beef/pork in India could not increase due to religious and cultural norms). Desirability, defined here in terms of the cost of giving up certain preferred foods, uses a measure developed by Irz et al. (27, 56) as a monetized metric of the disutility experienced by consumers when shifting to less preferred foods. Even though this was expressed in monetary terms, it is a social outcome based on preferences linked to taste rather than an economic outcome.

It is important to note that of the paucity of outcomes captured under the social pillar, two thirds were focused explicitly on consumers. Frehner et al. (57) were the only team to explicitly incorporate a supply chain lens, using an aggregated measure that did not differentiate risks to different groups of stakeholders. Notably absent from the measures were any outcomes focused explicitly on the well-being of workers or animals.

### 4.4. Use of valuation measures

Six publications used economic valuation techniques to assign monetary values to one or more outcomes included in our pillar definitions. As with the desirability (or cost of changing tastes) measure discussed above, such outcomes were assigned to the pillars to which they corresponded based on the underlying phenomena measured, not the units reported (e.g., dollars, euros). For example, Springmann et al. (9) reported the social cost of carbon as an estimated outcome. Although the unit of analysis was USD trillion, this outcome was captured in the “Climate Change” category in Table 3, as the authors estimated the societal impacts associated with climate change. Similarly, Broeks et al. (58) assigned a value of €50,000 and €100,000 per QALY gained to measure the impacts of an introduction of a meat tax or fruit and vegetable subsidy. Despite the monetary valuation, this outcome was assigned to the QALY/QOL category. Overall, 15 environmental pillar outcomes, 5 health pillar outcomes and 2 social pillar outcomes used valuation techniques.

### 4.5. Geographies

**FIGURE 4:**
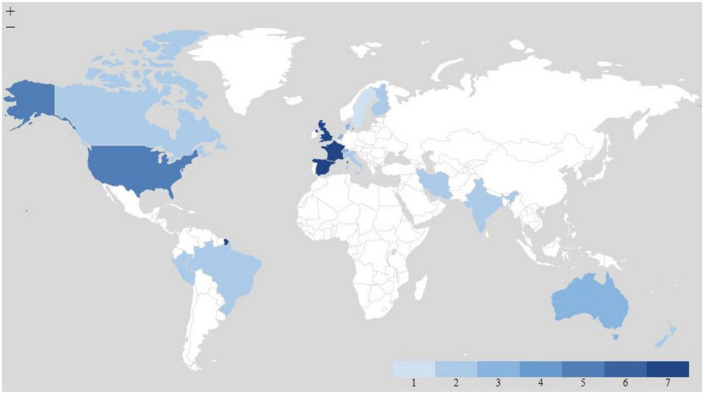
Geographic distribution of study country or region. This map does not include one European study and four global studies.

The most represented countries in this review are Spain (N = 6), France (N = 6), the United Kingdom (N = 6), and the United States (N = 4). Low- and middle-income countries (LMICs) remain under-represented in research on healthy diets from sustainable food systems. Of the 42 publications included in this review, only six are explicitly focused on LMIC contexts (Figure 4). These countries included Lebanon, Brazil, India, Iran, and Peru – all middle-income nations rather than low-income. This can be explained in part due to the lack of datasets from other parts of the world, early proof-of-concept analyses using available data that was initially derived from high-income settings, and a greater focus in such countries on health outcomes for which data are plentiful (i.e., NCDs, all-cause mortality, DALYs, etc.). Many analyses often claim to have “global” implications; however, diets and disease patterns vary significantly in rural Africa or South Asia when compared with the rest of the world.

## 5. Discussion

The literature linking dietary patterns to human and planetary health has expanded rapidly. A growing number of analyses integrate interactions across multiple domains, with a rising frequency of publications in this field. That said, based on our pillar definitions (Table 1), this review suggests that there remains a paucity of studies that collectively integrate data from all four major pillars into a systematized analytical framework. Indeed, 41 publications were excluded from this review for not addressing more than two diet pattern-outcome dynamics, despite the appearance of key search terms in the title and/or abstract suggesting otherwise.

### 5.1. Study findings are converging

There is broad consistency in several conclusions emanating from research published since 2015, and much of that is coherent with earlier publications. Among major findings there is broad agreement that a reduction in and/or substitution of animal source foods with plant foods in high-income country diets could have important beneficial impacts on human health, climate change emissions, and natural resource use. A second area of convergence is that current average dietary patterns are deemed to be unsustainable in both environmental and health terms. As a corollary, shifting consumption patterns, particularly in high- and middle-income countries, to align better with national food-based dietary guidelines would generate both environmental and health benefits, simultaneously. Third, there are important tradeoffs across different policy goals when it comes to shifting dietary patterns, and it is perhaps impossible to achieve optimal benefits across all domains. On the one hand, tradeoffs may occur across environmental parameters, with, for example, a shift to plant-based from animal-sourced foods reducing GHG emissions but resulting in higher uptake of water. On the other hand, net environmental benefits may accrue more with some diets than others, and optimal human health outcomes will vary too.

### 5.2. Modeling costs of alternative diets is common, but more critique of assumptions needed

Some estimates conclude that there would be no increase or even a decline in the cost of alternative diets, as in Switzerland (59) and Spain (60). One global simulation model (4) found that diets of higher nutritional quality, that were also less environmentally damaging, would cost less than current diets in many high-income settings; the reduction in cost was mainly attributed to lower spending on animal-source foods. In contrast, attaining a healthier diet in low-income settings would likely carry higher costs than today – mainly because a greater quantity and variety of foods would need to be consumed than is currently being consumed (4). The latter general conclusion has been replicated by several other researchers. For example, using a different computable general equilibrium (CGE) model, Philippidis et al. (61) also concluded from their analysis of 140 countries and regions that the EAT-Lancet Planetary Health Diet would result in an increase in marginal food expenditures per capita for resource-constrained populations. Yet, Goulding et al. (62) determined that wide adoption of the Planetary Health Diet in Australia would be more affordable for all socioeconomic groups than the Typical Australian Diet basket.

In some cases, the net benefit economy-wide (versus accruing at the household level), required that lower spending on food (in Scotland) associated with less meat consumption be spent locally to generate economic multipliers other than reduced healthcare spending (63). The benefit could also be different by country. Modeling by Irz et al. (27) on diet shift impacts on health and climate in Europe showed that trade-offs are common and that unexpected outcomes result from cross-food substitutions. Finally, of the publications included in this review, none were designed to take on more than 3 of the 4 pillars (as defined by our criteria) necessary to fully understand how the dietary choices of individuals impact the world around them. Thus, the oft-mentioned goal of enhancing analyses of the health and environmental sustainability of diets with an understanding of economic costs and benefits, and social justice and equity parameters, remains elusive. More attention is needed to articulating assumptions on methods and tradeoffs, particularly when seeking to incorporate cost externalities (impacts of food system functions underpinning different diets on, for example, environmental parameters and future health care).

### 5.3. Priority knowledge gaps

Issues emerging from this review suggest a set of five important needs. First, greater transparency and clarity in methods is needed in all future publications in this field to better allow for comparison and replication. For example, whether LCA or other approaches are used to determine the impacts of dietary patterns, there is variability in how boundaries are set for such analytical work. Indeed, there is often a lack of transparency about elements that are *not* included in analytical frameworks. This can be seen in how emission estimates often do not include activities beyond the farm, most notably transportation (64). Such system boundaries inevitably impact results of studies. This effectively truncates analyses and prevents a full assessment of system-wide impacts of dietary choices and alternatives. The need for transparency, fuller articulation of assumptions, and better justification of choices is noted in many of the publications retained for this review.

The lack of space allowed in most peer-reviewed publications severely limits textual elaboration regarding data quality, methods used in integrating disparate sources of information into complex models, and choices made by the authors regarding selection and use of some indicators but not others. Too many of the papers reviewed posed significant challenges to the reader in seeking to interpret findings and the resulting narrative. Béné et al. (65) highlighted the paucity of conceptual clarity when multiple dimensions and indicators have been proposed and/or used to characterize how diets interact with the many facets of ecological and socioeconomic systems.

Second, more explicit integration of metrics linking both social and economic issues to those of more conventional diet-climate-planetary ecology relationships is needed. The number of metrics used to assess human and planetary impacts of dietary choices has expanded but remains centered around diet-related non-communicable disease states, on the one hand, and GHG emissions and a range of natural resource depletion and/or pollution outcomes on the other hand. The small number of results for all other recorded outcomes is likely a function of data availability. Many outcomes (e.g., eutrophication and biodiversity) are spatially dependent and require more complex data development and modeling using LCA for estimation than modeling climate or land use.

When it comes to economic and social concerns, the range of suggested metrics continues to grow but data availability, validation, and effective incorporation into modeling remain challenging. It is necessary to prioritize building our understanding of the social and economic dimensions of sustainability to address food security and nutrition both equitably and affordably (14). The findings generated from this review concur with that conclusion and push the boundaries further to include social sustainability along the whole value chain, and not just for consumers.

The range of metrics referred to in the literature on social sustainability is growing steadily, despite not being systematically built into complex modeling. Many recent publications mention the need for incorporating ideas of equity, decent work and social justice into the conceptualization, measurement and policy frameworks for food system transformation (30, 66, 67). Example definitions of social metrics include gender equity, child labor, community rights, farmer’s livelihood, working conditions and worker wellbeing, animal welfare, female labor force participation, and livable wages for workers (7, 13, 65, 68–70). However, very few of these metrics can be incorporated into analyses due to a lack of data. Child workers, women, migrants, and undocumented workers are often concentrated, and sometimes even hidden from oversight, in poor quality jobs where they are most vulnerable to unjust practices. Thus, it is difficult to capture their experiences in meaningful metrics at comparable scales to the other pillars. That said, new research that estimates one labor metric−risk of forced labor−at the food-level has emerged in the US (71), which shows promise for integration into future diet sustainability studies.

The literature reviewed here lacks specificity when it comes to their references to social concerns, and even fewer include social metrics in their formal analyses. Most studies claiming to address social issues relied on disaggregating results based on conventional measures such as socioeconomic status, education level, or race. Disaggregation of results by subpopulations within countries is an important and underutilized approach to analyze social inequities across populations in the diet sustainability literature. Nonetheless, this approach does not describe social outcomes. There is a strong need for greater attention to both metric creation for social outcomes of diets and sub-national analyses that disaggregate results by social groups.

Given how little space is typically dedicated to the reasoning behind selection of metrics, the basis on which various indicators have been selected or applied are frequently unclear. All too often, it appears that indicators are used because they are available and have some degree of resonance when applied to one domain versus another, rather than based on an analysis of potential alternatives.^1^ The right choice of metrics is key to understanding causal mechanisms, to assess the potential impacts of food system transitions, and to identify cost-effective policies at scale. This review suggests that the field requires more creativity and complexity rather than standardization of metrics or approaches, but in allowing for that much greater exposition is needed on assumptions, particularly those relating to system boundaries applied to the analytical approach, data limitations, sensitivity analyses on the effects of methodological choices, and interpretability of results.

Third, more case studies relating to low- and middle-income countries and to broader regions should be conducted. Low- and middle-income countries remain under-represented in this literature, which tends to focus on the global level or at national studies for mainly high-income countries. The geography of food matters in terms of the characterization of local diets. Steenson and Buttriss (16) call for use of less generic “data sources to estimate current and alternative dietary intakes, nutritional content of foods, bioavailability of nutrients from these foods and associated environmental and health impacts,” in part because the climate and ecological impacts of production technologies and practices also vary. For example, extensive pastoral production of camel’s milk in Somalia likely has different GHG emissions than intensive dairy farming in Germany. Furthermore, livestock in smallholder systems often provide services in addition to products (i.e., drought power and asset value), which can dramatically reduce estimates of environmental impact per unit product when included (72). This is usually unaccounted for in environmental LCA analyses of diets. Therefore, the realities of international food trade also come into play when determining context-specific environmental, social, and economic externalities of food production. As a result, some papers highlight the need for much more analysis relevant to LMICs, but so far, the response to such calls has been very limited.

At the same time, the technical assessments on which so many studies rely when calculating emissions and resource use, assume universal applicability of data derived almost exclusively from high-income settings. Indeed, more broadly, most studies reviewed relied on assessments published in earlier literature rather than calculating their own relationships, which can present problems. The lack of standardized LCA methodologies and LCA databases to accurately represent marketplace foods has led to variability across studies, which can result in inconsistent recognition of life cycle stages and scales, thereby underestimating GHG emissions and limiting comparisons across studies (12, 14, 73).

Fourth, there is a need for greater inclusion of processed foods in assessment of diets to reflect the reality of consumer choices globally. In considering types of foods by context, it is also important to note that most of the literature relies on assessments of the environmental and health externalities of foods assessed as “whole commodities” (such as meat or dairy or cereals or fruits). There is still too little understanding of the environmental, health and social impacts of food choices that are most common in urban settings around the world – food items that are processed and packaged food items in various forms. Conventional boundary-setting that limits attention to food system functions post-farmgate leads to the exclusion of widely consumed items, such as ultra-processed foods and sugar-sweetened beverages (SSB). This can present a major limitation in studies looking at impacts of diets. Indeed, one study has found that ultra-processed foods has represented up to 15% of total kcal/day for some high-income earners (53). The water, energy use and emissions relating to production, transportation, retail, and consumption of SSBs, cakes, pastries, pies, sweets, and fried foods can be as significant as their health implications, yet few analyses have incorporated a fuller basket of foods in their dietary assessments.

Also important is the contextualization of diets. Often, food consumption is a product of multiple factors, including nutritional quality, enjoyment, taste, and as a means of participating in socio-cultural aspects (24). In other words, the cultural dimension of meals matters beyond sufficing nutrient needs, but this aspect has been factored into diet optimization studies in a very limited capacity (54, 55). Cultural acceptability is often defined as how food consumption in a proposed diet deviates from observed consumption in a region. This operationalization is insufficient, as there is little consideration to the nuances associated with cultural and traditional consumption that is native to a region or country. These cultural consumption patterns may not be as apparent when looking at the observed dietary patterns for a region but are still of value and very much part of food choices within subregions. Therefore, further exploration and development of metrics to measure cultural acceptability that takes into consideration traditional foods as well as foods important to different regions is imperative.

There are sex and age dimensions that also need to be considered. Where food impacts on health are a priority concern, average consumption patterns will mask divergence in terms of access; that is, the socioeconomic dimensions relating to inequitable income, purchasing power and physical proximity to markets, as well as in terms of outcomes for individual consumers. Most studies analyze data from men and women combined and provide estimates of the mean effect of diet on these pillars. Yet, Strid et al. (74) in considering the impacts on health of diets in Sweden by high versus low GHG emissions found different sex-specific outcomes, arguably because the nutrient needs of men and women differ, and their food preferences may also vary.

Fifth, greater attention should be paid to the implications of findings for policymakers (including ensuring their ability to appropriately interpret results). In terms of policy relevance of scientific outputs in this field, Peng et al. (75) argued that while today’s more complex integrated assessment models combine insights from climate science and economics to estimate how food system actions might support transformative action to tackle global warming, these models are theoretical and fail to encapsulate the cross pressures of trade-offs politicians face. For example, if crop yield is sacrificed to reduce environmental impacts of high-input farming in one location or country, then the market will likely incentivize more intensive farming in other places, potentially using lower standards for environmental governance and more negative impacts on working conditions of agricultural laborers. Similarly, studies from Sweden have shown that consumers choosing diets known to have low GHG emissions had higher intakes of added sugar (because sugar is a plant-based product with low climate impact but a known human health risk), than people choosing foods with high diet-related GHG emissions (74, 76).

## 6. Strengths and limitations

This study has important strengths. We used a standardized, rigorous approach to review the current literature on integrated sustainability assessment of dietary patterns. Furthermore, we operationalized a typology of dietary patterns to discern differences in how this field undertakes analyses of sustainable diets. In the same vein, we operationalized the four pillars to be both explicit and outcome focused. For the social pillar, we explicitly include stakeholders along the value chain beyond consumers, thereby linking relevant frameworks from social life cycle assessment (S-LCA) to the food systems field (45). Our strict operationalization of the pillars to be outcome focused, especially for the health pillar, provided clarity. Many studies in the literature indicate they analyze health outcomes, but instead focus on indicators like diet quality measures, which may be associated with some health outcomes but are not outcomes in themselves. Our strict definitions could be interpreted as both a strength, and a limitation as they limited the scope of included papers, likely significantly.

A limitation of our review is that we were restricted to including a relatively small number of publications which can be ascribed partly to the novelty of research questions being asked and the strict application of inclusion criteria intended to minimize bias, and our own definitions of the pillars of concern. Publications were excluded if they did not pair dietary patterns with at least two pillars of interest, were not in the English language, and if they applied methodological approaches that did not allow for direct comparison among studies, such as, if only parts of the diet were analyzed. Because of the few studies, and the variation in study descriptors and analysis, we were not able to do a meta-analysis of data or report on the bias/limitations of the existing research. Our summary of the data is in line with evidence gap mapping (77), and is designed to identify gaps in the research.

## 7. Conclusion

More than half a decade ago, Jones et al. (78) argued that “a lack of clear metrics and a shared approach to measuring the multiple components of sustainable diets has hindered progress toward generating the evidence needed.” That statement holds today. We need progress. While “the evidence base remains incomplete and constantly evolving” (16), there are gaps in metric use, analyses and choices that should be prioritized for future research. Such gaps center mainly on questions of social justice and how to best measure and model them in relation to dietary patterns, but also to questions of how the local cost of different diets might change under a range of assumptions and scenarios. That said, diets are underpinned by the characteristics of local food systems, and much more needs to be understood about the cultural aspects of dietary choices as well as the drivers of different food prices which operate across entire food value chains, not just in the retail or institutional food environments which dominate thinking about consumer choice.

There also remains significant heterogeneity across studies in terminology, definitions, and datasets used. Data availability typically drives the metrics used in such studies. Béné (43) highlighted “the generally incomplete, fragmented and static datasets that we have at our disposal at present,” while Thornton et al. (79) “encountered severe challenges and limitations because of lack of data and lack of comparability across different information sources.” This makes it difficult to generate rigorous comparable evidence needed to guide and cost policy recommendations, set targets, track progress, and assess what works. The paucity of data and related analyses focused on low- and middle-income country contexts represent major constraints to understanding truly global patterns and trends, but also to identifying appropriate policies and strategies outside of high-income settings.

There are significant methodological challenges to be faced in seeking to fully integrate all four of the fundamental facets of sustainability where diets link human and planetary health. This scoping review identified no papers that included measures relevant to environmental, health, economic and social concerns simultaneously. Researchers must move toward novel, more complex frameworks that make looking across a variety of measures across all four pillars commonplace. A continued focus on simple domain pairings (like diet and climate) using a small number of metrics that often only proxy outcomes of interest, standardized comparisons among studies will continue to be challenging, and specific recommendations about how to manage uncertainty as well as trade-offs will remain vague. Innovative, interdisciplinary methods are needed to standardize and weigh relative impacts, thereby to provide context-specific and actionable policy as well as consumer guidance.

New approaches, such as “true cost accounting” frameworks that seek to price-in environmental and human externalities to the cost of dietary patterns, are still too-often poorly defined and measured. Such complex multifactorial analyses need to support more insightful conclusions that can contribute to coherent narratives of change. Future target-setting and policy negotiations aimed at food system transformation deserve to be better informed about potential scenarios, relative costs and benefits, likely tradeoffs, and multiple-domain impacts – with a much deeper understanding of the implications of alternative dietary patterns at the core.

## Data availability statement

The original contributions presented in this study are included in the article/Supplementary material, further inquiries can be directed to the corresponding author.

## Author contributions

PW and NB: conceptualization. KL, HL, BH, KB, and BB: extraction. PW, BH, KB, JM, BB, and NB: writing—original draft and revise manuscript. KL, NM, SC, FZ, and JD: writing—review and editing. BB, BH, KB, and JM: data curation. BB and JM: visualization. PW, NB, and KL: supervision. All authors contributed to the article and approved the submitted version.

## Footnotes

1 Melesse et al. (70), limited themselves in the name of “practical applicability” to “indicators for which data is covered in datasets widely available for low- and middle-income countries,” while Béné et al. (80) only used indicators “that are publicly available for all countries.” While these represent pragmatic and defensible choices, the indicators in public datasets may not in fact be the most appropriate to use for complex analyses.
