# Supplementary material for: Measurement of diets that are healthy, environmentally sustainable, affordable, and equitable: A scoping review of metrics, findings, and research gaps
Source: Front Nutr. 2023 Apr 3;10:1125955. doi: 10.3389/fnut.2023.1125955 (PMC10106581; doi:10.3389/fnut.2023.1125955)
Supplement: Supplementary file 1 [file Table_1.DOCX]

Supplementary Material

Measurement of Diets that are Healthy, Sustainable, Affordable and Equitable: A Scoping Review of Metrics, Findings, and Research Gaps

Patrick Webb^1^*, Kara Livingston Staffier^2^, Hyomin Lee^1^, Brian Howell^1^, Kyra Battaglia^1^, Brooke M. Bell^1^, Julia Matteson^1^, Nicola M. McKeown^3,1^, Sean B. Cash^1^, Fang Fang Zhang^1^, Jessica L. Decker Sparks^1^, and Nicole Tichenor Blackstone^1^

*** Correspondence:** Patrick Webb: Patrick.Webb@tufts.edu

# Supplementary Table

**Table A1. Search term strategy**

| **Pillars** | **Search Queries** |
| --- | --- |
| Diet | ((diet* or eating or food) NEAR/3 (choice* or choos* or pattern* or habit* or decision* or price or cost)) or "diet quality assessment" or "diet quality index" or "diet pattern analysis" or "diet* quality" or "quality of diet" or "diet* pattern" or "diet* choice" or "sustainable diet*" or "sustainable food consumption" or "sustainable nutrition" or "ecological diet*" or "resilient diet*" or "biodiverse diet*" or diet* or dietary pattern or dietary change or dietary recommendation or eating pattern or food pattern or “food intake” |
| Environment | ((climat* or environment* or sustain* or carbon or "greenhouse gas*" or "planetary warming" or ecological* or "natural resource*" or renewable* or "non renewable*" or nonrenewable*) NEAR/3 (impact* or effect* or cost* or ramification* or implicat* or footprint* or harm*)) or "environmental impact" or "environmental footprint" or "environmental assessment" or "climate change" or "greenhouse gas" or "land use" or "water use" or "carbon footprint" or "life cycle assessment" or sustainability or "planetary bound*" or "climate change" or "global warming" or "greenhouse gas" or "carbon footprint" or nitrogen or water or "land use" or "energy use" or "biodiversity" or "environmental impact" or pollution or “planetary boundary” or “greenhouse-gas emission” or “carbon footprint” or “environmental impact” or “planetary health” |
| Health | "noncommunicable disease" or "risk factor" or "health outcome" or "nutritional quality" or healthy or "risk factor" OR "disease outcome" OR malnutrition OR "~nutrient deficiency" OR "noncommunicable disease" OR "chronic disease" OR obesity or "health outcome" OR morbidity OR mortality OR "cardiovascular disease" or "disease outcome" or "health outcome" or malnutrition or "noncommunicable disease" or "chronic disease" or "relative risk" or daly or qaly or "risk factor" or micronutrient or "type 2 diabetes" or stunting or wasting or overweight or obesity or “global burden of disease” or haly or morbidity or mortality or "noncommunicable disease" or "cardiovascular disease" or “nutritional status” or “global health” OR “human health” |
| Economic | ((econ* or value or agricultur* or producer* or farm*) NEAR/3 (pricing or price* or cost* or chain* or livelihood* or afford* or income* or external*)) or econometric models or econometrics or economic analysis or economic evaluation or economic theory or economic impact or cost effectiveness analysis or economic impact or “food price” or “farm price” or “diet price” or “diet cost” or “cost of production” or income or livelihood* or profit or “value chain” or cost or “true cost accounting” or “hidden cost” or "econometric model" or "economic analysis" or "economic impact" or "economic evaluation" or "cost effective analysis" or "food price" or "diet cost" or “full cost accounting" or "full price accounting" or "cost analysis" or “Equilibrium Modeling” or “opportunity cost” |
| Social | "social impact" or "social justice" or "livable wage" or "socioeconomic status" or inequity or disparit* or "farm labor" or "farm worker" or "social perspective" or "social sustainability" or "social equity" or "social dimension" or "social indicators" or "development sustainability" or free lab?or or forced lab?or or child lab?or or abuse lab?or or exploit* lab?or or work* condition* or migrant worker* or food worker* or rights or wage or livable wage or underpayment or farmworkers or justice or injustice* or "social welfare" or "human right*" or "social cost*" or "equal rights" or "social outcome*" or equity or equitable or inequit* or "unequal outcome*" or disparit* or "common good" or equity OR disparity OR injustice OR "living wage" OR "worker's right" OR "socioeconomic status" or farmworkers or justice or injustice* or "social welfare" or "human right*" or "social cost*" or "equal rights" or "social outcome*" or equity or equitable or inequit* or "unequal outcome*" or disparit* or "common good" or justice or injustice* or "social welfare" or "social cost*" or food security or "equal rights" or human right* or equit* or inequit* or disparit* or minorit* or “forced labo$r” or “child labo$r” or “free labo$r” or ((justice or injustice* or "social welfare" or "social cost*" or food security or "equal rights" or human right* or equit* or inequit* or disparit* or minorit* or labo?r) NEAR/2 (free or forced or child)) |
